# Hidden diversity in waterfall environments: The genus *Acrorbis* (Gastropoda: Planorbidae) from the Upper-Paraná Atlantic Forest

**DOI:** 10.1371/journal.pone.0220027

**Published:** 2019-07-19

**Authors:** Roberto E. Vogler, Alejandra Rumi, Leila B. Guzmán, Ariel A. Beltramino, Enzo N. Serniotti, Walter Ferrari, Juana G. Peso

**Affiliations:** 1 Instituto de Biología Subtropical, CONICET—Universidad Nacional de Misiones, Posadas, Misiones, Argentina; 2 División Zoología Invertebrados, Facultad de Ciencias Naturales y Museo, Universidad Nacional de La Plata, CONICET, La Plata, Buenos Aires, Argentina; 3 Centro de Estudios Parasitológicos y de Vectores (CEPAVE), Facultad de Ciencias Naturales y Museo, Universidad Nacional de La Plata, CONICET, La Plata, Buenos Aires, Argentina; National Cheng Kung University, TAIWAN

## Abstract

High-energy freshwater environments such as rapids and waterfalls in the Upper-Paraná Atlantic Forest are home to highly endemic minute freshwater snails of the genus *Acrorbis*. Only one species, *Acrorbis petricola*, is currently included within this genus, whose geographical distribution is restricted to three known populations, one in Brazil and the other two in Argentina. Because of habitat specificity and limited geographical distribution, the species is considered vulnerable in Argentina and endangered in Brazil. In this work, we identify five new populations of *A*. *petricola* in southern Upper-Paraná Atlantic Forest, exclusively found on waterfalls from the Misiones Province, Argentina. Based on these populations and on specimens of one of the two historical populations from the Misiones Province, we explored the morphological features of shells and reproductive system of specimens from each location and provide the first molecular data on the species. We used DNA sequences from *cytochrome c oxidase subunit I* (*COI*) and *16S-rRNA* genes to investigate the molecular diversity, genetic distances and genealogical relationships among populations. We verified the existence of intra- and interpopulation morphological variability, with the greatest variation being found in spire, spiral sculpture, penis sheath, flagella, prostatic diverticula and bursa copulatrix. We found interpopulation genetic diversity, with no intrapopulation variation, and identified six geographically structured genetic lineages with maximum genetic distances of up to 2.3%. Different combinations of morphological characters with the same genetic background within each locality were observed. The finding of new populations genetically differentiated not only broadens the known distribution of the species, but also illustrates that waterfall environments in the Atlantic Forest harbour a hidden diversity of *Acrorbis* that still remains to be discovered. This scenario suggests a complex evolutionary history that needs to be unveiled and taken into account for future development of conservation strategies in this endemic genus.

## Introduction

Waterfalls and river rapids are natural laboratories for evolutionary research as they usually host a highly endemic flora and fauna, especially adapted to the constant humid conditions and the force of water, including several molluscan species [1–4]. Such high-energy freshwater environments are abundant in the Atlantic Forest, which extends across southeastern Brazil, eastern Paraguay and northeastern Argentina [5,6].

The Upper-Paraná Atlantic Forest, the southwestern and largest ecoregion of the Atlantic Forest, constitutes one of the “hottest hotspots” of biodiversity and endemism worldwide [5,7]. Despite ongoing landscape transformation, the Misiones Province in Argentina currently contains the largest remaining tract of this ecoregion [6,8], where numerous streams and rivers still maintain complete native species assemblages, including a high species richness of freshwater gastropods [9–11]. Nonetheless, in spite of being one of the most explored areas at malacological level, the molluscan biodiversity inhabiting waterfalls and rapids in the Upper-Paraná Atlantic Forest remains largely unknown [1,12–14].

Snails of the genus *Acrorbis* Odnher, 1937 (Gastropoda: Planorbidae) represent a clear example of endemic micromolluscs occurring in poorly studied high-energy freshwater habitats of the Upper-Paraná Atlantic Forest, for which currently available knowledge is scarce and fragmentary. Odhner [15] introduced the generic name *Acrorbis* to accommodate *Acrorbis petricola* Odhner, 1937, an atypical planorbid described from specimens collected in rocky banks of the Ariranha River, Nova Teutônia, Santa Catarina State, Brazil [15–20]. Under the name *Acrorbis odhneri* Hylton Scott, 1960, a new species was added to the genus from Argentina based on specimens collected in Salto Encantado, a waterfall located in the Salto Encantado Provincial Park, Aristóbulo del Valle municipality, Misiones Province [21–24]. Subsequent studies based on shell, anatomy and radular morphology of the species from Salto Encantado, verified that Argentinian specimens were indistinguishable from the Brazilian congeners, and consequently *A*. *odhneri* was regarded as a junior synonym of *A*. *petricola* [22]. A new comparative study in the 1990s, based on the finding of *Acrorbis* individuals in the Iguazú National Park, Misiones Province, Argentina, showed some variability in shell morphology and male terminal genitalia in relation to previous descriptions [24]. Nonetheless, based on the Iguazú specimens and on a reexamination of the type-series of *A*. *odhneri*, that study also suggested that both nominal species were conspecific under the name *A*. *petricola* [24].

To date, *Acrorbis* is considered to be monotypic [9–11,14,22,24,25] and is known only from a geographical area restricted to three localities in northeastern Argentina and southern Brazil: Salto Encantado Provincial Park (Salto Encantado waterfall) and Iguazú National Park (Salto Arrechea, Salto Dos Hermanas and Salto Rivadavia waterfalls), both in the Misiones Province, and Nova Teutônia, Santa Catarina State, its type locality in Brazil [14,24–26]. The species has always been found associated with high-energy freshwater environments in a landscape dominated by subtropical forest. The specimens seem to have restricted microhabitat preferences, such as the water–air interface on the surface of rocky substrates covered by moss and epilithic algae in the area kept wet by the water [1,22,24,26]. Owing to its limited distribution and niche specificity, the species is recognized as vulnerable in Argentina and endangered in Brazil [11,27]. However, no conservation strategies have been adopted for this species yet.

In this study, we surveyed waterfall environments in central and southern Misiones Province, Argentina, and identified new *Acrorbis* populations occurring at the Upper-Paraná Atlantic Forest. The objectives of this paper are: (i) to confirm the taxonomic identity of the populations using traditional morphological criteria based on shell morphology and morphological features of the reproductive system; (ii) to investigate the morphological variability among localities; and (iii) to explore the genetic background of the populations and to provide the first molecular data for the species. In this process we gained insights into a hidden diversity of *Acrorbis* in waterfall environments of the Atlantic Forest.

## Materials and methods

### Snail collecting and preservation

Living snails were collected by hand in six waterfall environments in central and southern Misiones Province during mollusc surveys conducted between 2017 and 2018. The waterfall known as Salto Encantado in Salto Encantado Provincial Park was specifically surveyed as it represents the first historical record for *Acrorbis petricola* in the Misiones Province [21]. Permission for collection was granted by Ministerio de Ecología y Recursos Naturales Renovables de la Provincia de Misiones (Disp. No. 027/2018). Specimens were mostly collected among mosses and epilithic algae directly on the wet walls of the waterfalls, or on nearby rocks at the base of the waterfalls, in the area sprayed by water.

Living specimens were relaxed in water with menthol crystals for 30–60 min and immersed in hot water (80°C) for 30–60 sec. Soft parts were then separated from the shell and preserved in 96% ethanol for anatomical and molecular studies. Shells were firstly cleaned with a fine paint brush in a 10% sodium hypochlorite solution for 10 min to remove vegetation and other encrustations from external shell surface, and then washed with distilled water. Subsequently, shells were cleaned in isopropanol using an ultrasonic cleaner for 5–10 sec, and then air dried [28].

Voucher specimens were deposited in the malacological collection of the Instituto de Biología Subtropical (IBS-Ma), CONICET–Universidad Nacional de Misiones (UNaM), Misiones Province, Argentina. In addition, we obtained ethanol preserved tissues of museum specimens of *A*. *petricola* from Nova Teutônia, Brazil, and Iguazú National Park, Argentina, housed in the malacological collection at the Museo de La Plata (MLP-Ma; La Plata, Argentina). These samples were included herein in order to explore the genetic background of the other historical records of *A*. *petricola* in the Atlantic Forest, including its type locality.

### Morphological examination

**Fig 1:**
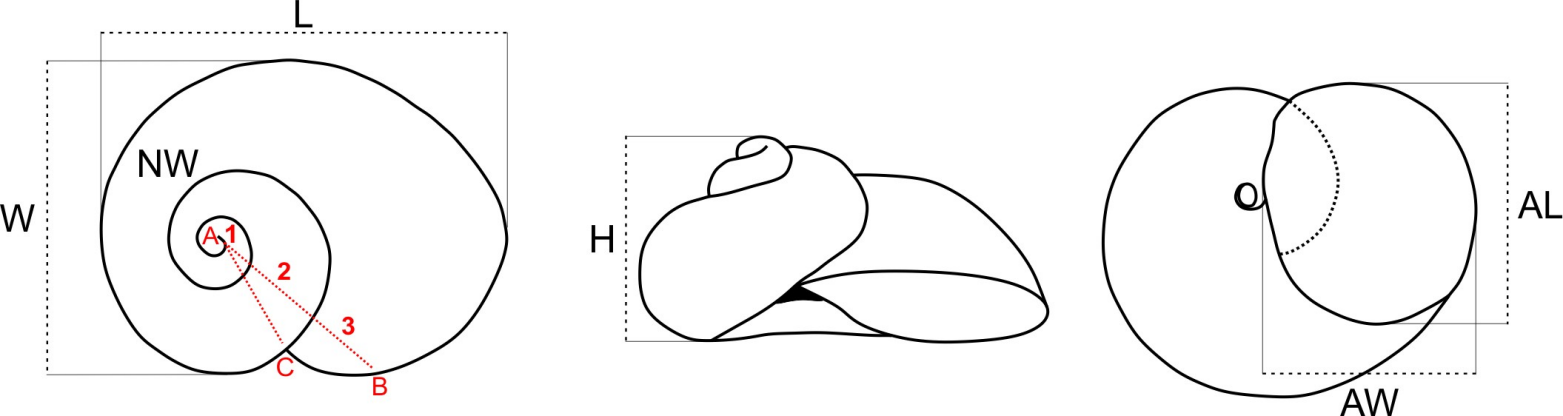
Scheme of *Acrorbis* shell measurements and whorl count. Red lines indicate the BAC angle for counting the number of whorls following Diver [29]. Abbreviations: AL, aperture length; AW, aperture width; H, height; L, length; NW, number of whorls; W, width.

Digital images in dorsal, ventral, and lateral views were obtained for ten specimens from each of the new localities with a Canon 6D camera equipped with a Nikon BD Plan 10X objective. Five shell measurements were taken in that material (Fig 1), namely the length (L = maximum dimension in the basal plane), width (W = maximum dimension perpendicular to L in the basal plane), height (H = maximum dimension perpendicular to the basal plane), aperture length (AL = maximum dimension of the aperture parallel to W, measured in the apertural plane), and aperture width (AW = maximum dimension of the aperture perpendicular to AL). Number of whorls (NW) was determined according to Diver [29]. Shells were measured using the software ImageJ 1.49 [30]. Shell measurements were normalized by logarithmic transformation and subjected to a principal component analysis (PCA) using PAST 3.25 [31] in order to explore the morphological variation among the different populations.

The anatomy of the reproductive system of three photographed specimens per locality was studied (voucher lots: IBS-Ma Nos. 376, 377, 378, 379, 380, 381). Specimens were dissected using a Labomed Luxeo 4D stereomicroscope and analysed according to Hubendick [18], Paraense & Deslandes [19], Paraense [20] and Ituarte [24]. When necessary, the structures were stained with 1% methylene blue solution or 0.1% neutral-red solution to enhance visualization [12].

### DNA extraction, PCR amplification, and DNA sequencing

DNA was extracted from 1 mm^3^ samples of foot muscle of ethanol-preserved individuals with an ADN PuriPrep-T kit (INBIO-Highway, Tandil, Argentina) following the manufacturer’s instructions. We selected 30 samples belonging to *A*. *petricola* (5 specimens per waterfall environment, including those anatomically dissected specimens) and the outgroup species *Antillorbis nordestensis* (Lucena, 1954) and *Biomphalaria peregrina* (d’Orbigny, 1835). Collection information and GenBank accession numbers for the samples analysed are presented in Table 1.

**Table 1:** Collection information and GenBank accession numbers for the samples analysed herein.

ID	Species	Geographical origin	Coordinates	Year	Voucher	Collector	GenBank Accession Nos.	
							*COI*	*16S*
1	*Acrorbis petricola*	Salto Encantado, Cuñá Pirú Stream, Salto Encantado Provincial Park, Caingúas Department, Misiones Province, Argentina	-27.05874 -54.82789	2018	IBS-Ma 376	Vogler R.E. Beltramino A.A. Guzmán L.B.	MK279672–MK279676	MK278863–MK278867
2	*Acrorbis petricola*	Salto Capioví, Capioví Stream, Parque Natural Don Alberto Nobs, Libertador General San Martín Department, Misiones Province, Argentina	-26.92463 -55.06197	2018	IBS-Ma 377	Vogler R.E. Beltramino A.A.	MK279677–MK279681	MK278868–MK278872
3	*Acrorbis petricola*	Salto Chávez, Acaraguá Stream, Campo Grande Municipality, Caingúas Department, Misiones Province, Argentina	-27.27199 -54.92287	2018	IBS-Ma 378	Vogler R.E. Serniotti E.N. Rumi A.	MK279682–MK279686	MK278873–MK278877
4	*Acrorbis petricola*	Salto Teodoro Cuenca, on a tributary of Del Medio Stream, Campo Ramón Municipality, Oberá Department, Misiones Province, Argentina	-27.40516 -55.01378	2017	IBS-Ma 379	Vogler R.E. Beltramino A.A.	MK279687–MK279691	MK278878–MK278882
5	*Acrorbis petricola*	Salto Krysiuk, Toro Stream, Guaraní Municipality, Oberá Department, Misiones Province, Argentina	-27.56978 -55.16169	2017	IBS-Ma 380	Vogler R.E. Beltramino A.A.	MK279692–MK279696	MK278883–MK278887
6	*Acrorbis petricola*	Salto Paca, Paca Stream, Panambí Municipality, Oberá Department, Misiones Province, Argentina	-27.68737 -55.00630	2018	IBS-Ma 381	Vogler R.E. Beltramino A.A. Serniotti E.N. Sotorres D.	MK279697–MK279701	MK278888–MK278892
7	*Acrorbis petricola*	Salto Arrechea, Arrechea Stream, Iguazú National Park, Iguazú Department, Misiones Province, Argentina	-25.65503 -54.45710	2005	MLP-Ma 14702	Rumi A. Núñez V. Ferrando N. Gutiérrez Gregoric D.E.	–	–
8	*Acrorbis petricola*	Nova Teutônia, Seara, Santa Catarina State, Brazil	-27.16542 -52.42502	2000	MLP-Ma 13791	Caldeira R.	–	–
9	*Antillorbis nordestensis* (Lucena, 1954)*	Puerto Península Lagoon, Puerto Península Provincial Park, Iguazú Department, Misiones Province, Argentina	-25.69410 -54.51377	2010	IBS-Ma 382	Vogler R.E. Núñez V. Gutiérrez Gregoric D.E.	MK279702	MK278893
10	*Biomphalaria peregrina* (d’Orbigny, 1835)*	Salto El Maynó, El Maynó Stream, San Vicente Municipality, Guaraní Department, Misiones Province, Argentina	-27.04649 -54.40040	2016	IBS-Ma 007	Vogler R.E. Molina M.J.	MK279703	MK278894

*Outgroup species.

Partial sequences of the mitochondrial *16S-rRNA* (hereafter *16S*) and *cytochrome c oxidase subunit I* (*COI*) genes were amplified using the primers 16SF-104 (5′-GAC TGT GCT AAG GTA GCA TAA T-3′) and 16SR-472 (5′-TCG TAG TCC AAC ATC GAG GTC A-3′) for *16S* [32], and the primers LCO1490 (5′-GGT CAA CAA ATC ATA AAG ATA TTG G-3′) and HCO2198 (5′-TAA ACT TCA GGG TGA CCA AAA AAT CA-3′) for *COI* [33]. PCR amplifications of the *16S* and *COI* genes were conducted as in Rumi et al. [34] and Vogler et al. [12], respectively, and run on a T18 thermocycler (Ivema Desarrollos). Success of PCR reactions was verified by agarose gel electrophoresis. Successfully amplified products were purified with an AccuPrep PCR Purification Kit (Bioneer, Daejeon, Korea). Owing to the co-amplification of nonspecific fragments, some PCR products were purified from 1.5% (w/v) agarose gel using an ADN PuriPrep-GP kit (INBIO-Highway, Tandil, Argentina). After purification, each gene was then directly cycle-sequenced in both directions (Macrogen Inc., Seoul, South Korea). Newly generated sequences and chromatograms were visualized and trimmed to remove the primers with Chromas Lite 2.6.5 (Technelysium Pty Ltd). The forward and reverse strands for each sequence were assembled by means of the BioEdit 7.2.5 software [35], with ambiguities checked and corrected manually. For the museum material of *A*. *petricola*, the repeated attempts to amplify *COI* and *16S* loci were unsuccessful; consequently, that material was not included in further analyses.

### Sequence data and phylogenetic analyses

Multiple sequence alignment of *16S* gene was performed using MAFFT 7 through the MAFFT web-server (https://mafft.cbrc.jp/alignment/server/) [36] with the G-INS-I algorithm and optimized by visual inspection. The *COI* sequences were aligned using Clustal X 2.1 [37], and translated into amino acids to check for stop codons and frameshift mutations in ORF Finder (https://www.ncbi.nlm.nih.gov/orffinder/). For *16S*, a putative secondary structure model was generated following the template proposed by Lydeard et al. [38] and contrasted with structural models for other molluscs available at the Comparative RNA Website [39]. This model was used to examine nucleotide substitutions in relation to conserved sequence motifs, alignment and secondary structure of domains IV and V of the *16S* gene predicted for other molluscs. The number of haplotypes (*h*), number of segregating sites (*S*), as well as nucleotide (π) and haplotype (*H*_*d*_) diversities per marker were computed by means of the DnaSP 6.12.01 program [40]. Nucleotide composition of haplotypes was analysed in BioEdit 7.2.5. Genetic distances were analysed in MEGA X software [41] using the number of differences (*p*).

Phylogenetic trees were constructed using maximum likelihood (ML), and Bayesian inference (BI). *Acrorbis* phylogenetic datasets were analysed separately as *16S* (279 bp), *COI* (655 bp) and concatenated (*16S* + *COI*, 934 bp). The ML analysis was conducted with PhyML 3.0 [42] via the ATGC bioinformatics platform (http://www.atgc-montpellier.fr/) with the Nearest-Neighbor Interchange branch swapping algorithm. Optimal models of nucleotide substitution were selected using the SMS program [43] by means of the Akaike Information Criterion: GTR+G model for all datasets. The statistical support of the nodes was evaluated using 1,000 bootstrap replicates [44]. For the concatenated dataset, we also conducted a ML analysis partitioned by gene with RAxML 8.2.12 [45] via the CIPRES portal (https://www.phylo.org/) [46], inferring with RAxML the appropriate parameters of the GTR+G model for each partition [47]. Nodal support was assessed using RAxML rapid bootstrapping with 1,000 bootstrap replicates. The BI was performed in MrBayes 3.2.6 [48] with the parameters from the best model (GTR+G for *COI* and concatenated datasets, and GTR+I for *16S* sequences) as identified in jModelTest 2.1.7 [49] under the corrected Akaike Information Criterion. Two runs were performed simultaneously with four Markov chains for 1 million generations. Trees were sampled every 100 generations, and posterior probabilities were obtained after discarding the first 1,001 samples of each run as burn-in. In addition, a median-joining network was constructed for each individual locus and the concatenated dataset using Network 5.0.0.3 [50], in order to visualise relationships among haplotypes.

## Results

### New localities records

**Fig 2:**
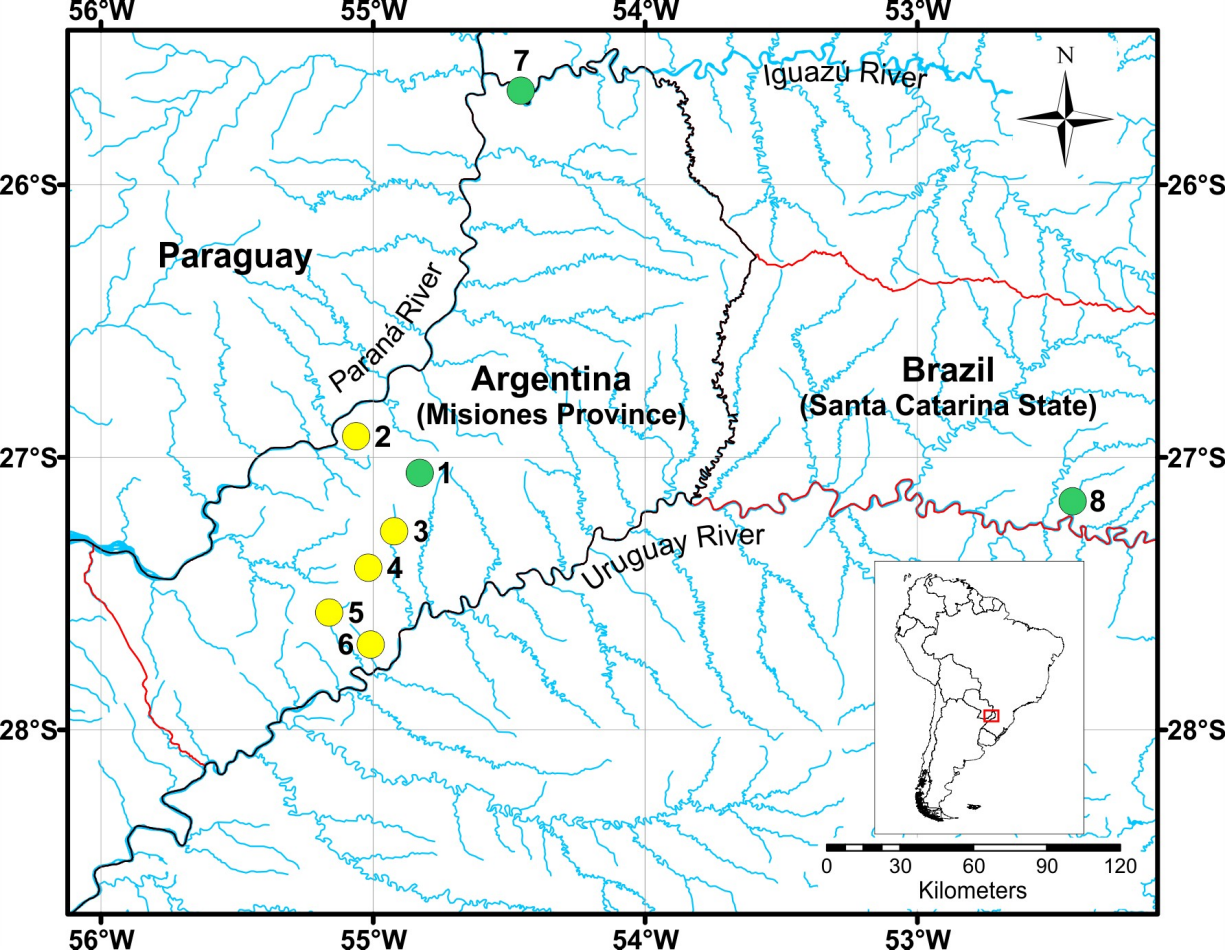
Distribution of *Acrorbis petricola* in the Upper-Paraná Atlantic Forest. Locations in green correspond to historical records and the yellow circles indicate the new localities where the specimens of *A*. *petricola* were found. Location numbers correspond to the numbers in Table 1. Type locality (No. 8).

**Fig 3:**
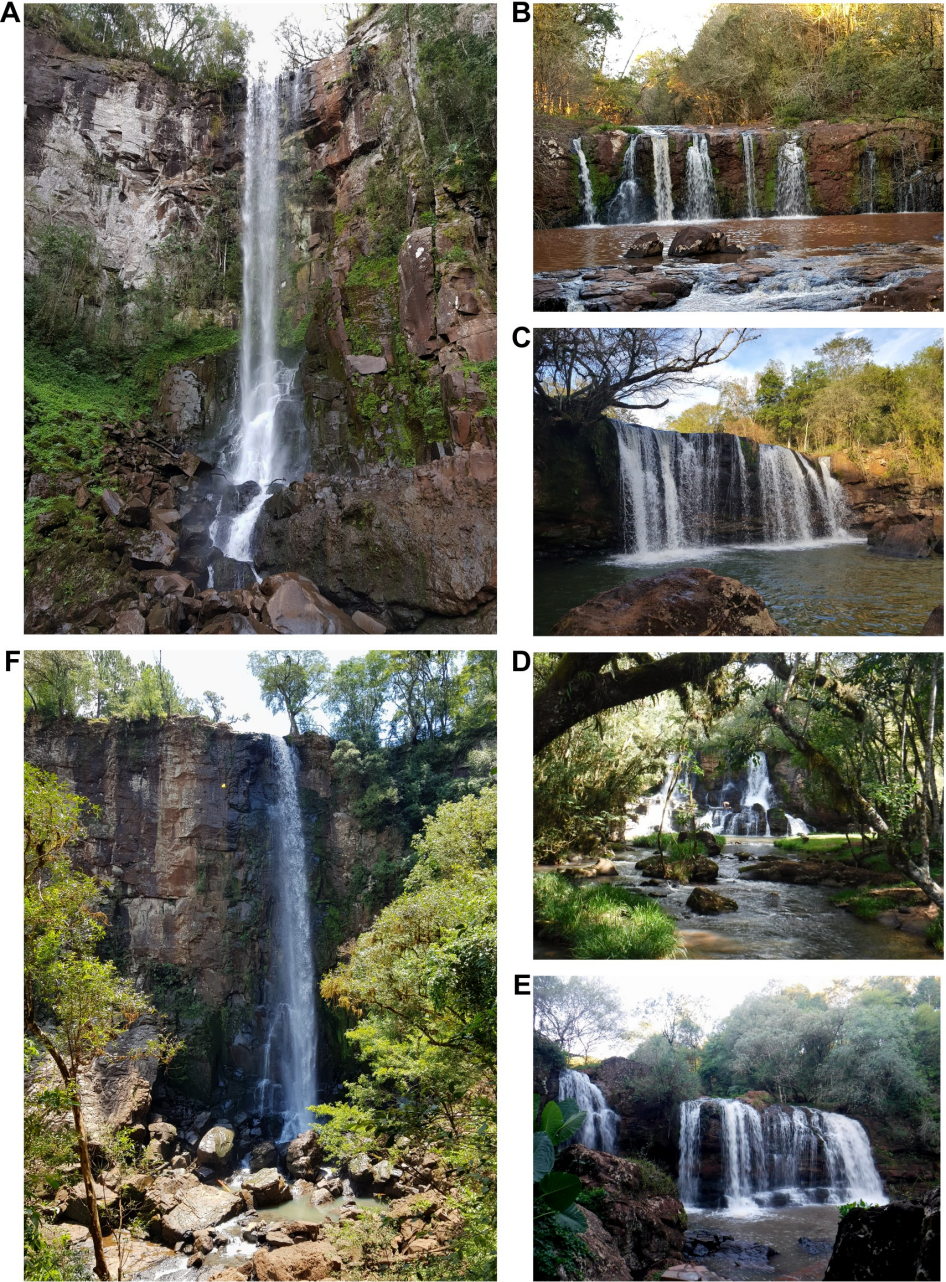
Waterfalls where *Acrorbis petricola* populations were found in central and southern Misiones Province, Argentina. A, Salto Encantado. B, Salto Capioví. C, Salto Chávez. D, Salto Teodoro Cuenca. E, Salto Krysiuk. F, Salto Paca. Coordinates to localities are provided in Table 1.

Living specimens of *Acrorbis* were recorded in the six waterfall environments surveyed in central and southern Misiones Province, five of these being new localities for the genus (Figs 2 and 3 and Table 1).

### Morphological examination

**Fig 4:**
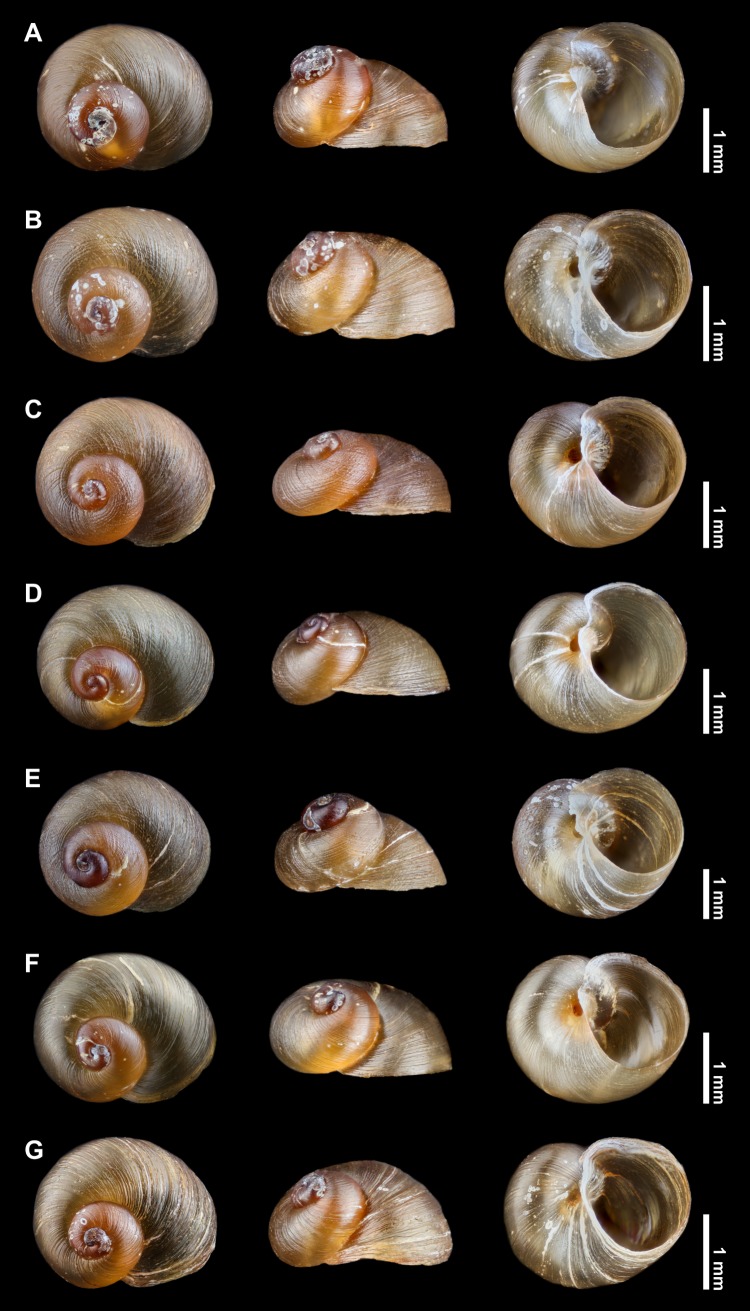
Shells of *Acrorbis petricola* from the Misiones Province in dorsal, lateral and ventral views. A, B, Salto Encantado (IBS-Ma 376–2 and IBS-Ma 376–8, respectively). C, Salto Capioví (IBS-Ma 377–5). D, Salto Chávez (IBS-Ma 378–1). E, Salto Teodoro Cuenca (IBS-Ma 379–3). F, Salto Krysiuk (IBS-Ma 380–3). G, Salto Paca (IBS-Ma 381–4).

**Fig 5:**
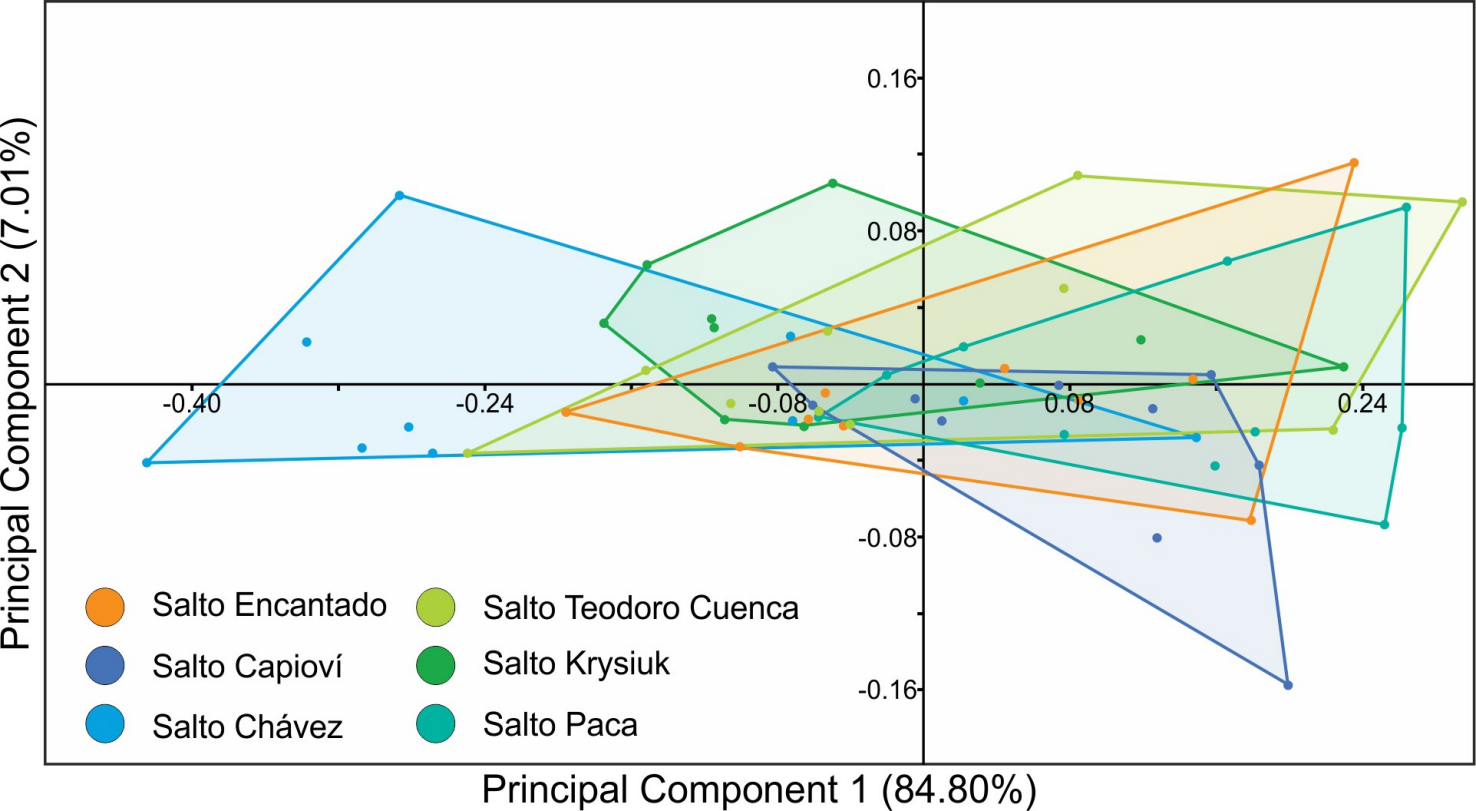
Scatter plot of the first two principal components (PCs) obtained by the principal component analysis (PCA) on specimens of *Acrorbis petricola* from the Misiones Province.

The specimens from the six waterfalls surveyed exhibited the morphological characters of shell and reproductive system defined for *Acrorbis petricola* as described by Odhner [15], Paraense & Deslandes [19] and Paraense [20]. The following distinctive characters are emphasized:

Shell: pseudodextral, small, helicoid; spire ranging from low and nearly depressed to elevated, with convex whorls increasing rapidly in diameter, up to 2 ⅞ in number in the largest specimens; suture deep (Fig 4 and Tables 2 and S1). Shell from pale amber to deep brown, apex deep brown, somewhat reddish in several specimens. Shell surface with oblique striae, some specimens sculptured with spiral lines from faint to coarse ones superimposed onto oblique striae giving them a somewhat reticulated appearance (e.g. Fig 4B and 4E). Apex laterally displaced, not the highest point on spire in several specimens. Last whorl strongly widening. Aperture wide, oblique, D-shaped to subcircular; lips simple, sharp. Most of specimens widely umbilicated with umbilicus partially covered by small basal reflection of inner lip, several specimens with umbilicus fully covered (Fig 4A). Average measurements of ten adult shells and the conchological variability recorded per waterfall environment are presented in Tables 2 and 3, respectively. In the PCA, the first two principal components explained cumulatively 91.81% of the total variation (PC1: 84.80%; PC2: 7.01%; S2 Table). There was no clear separation among the *Acrorbis* populations in the morphometric space, as all populations overlapped with at least one other (Fig 5).

**Table 2:** Average ± standard deviation and range (minimum and maximum values) of five shell measurements of *Acrorbis petricola* from six localities in the Misiones Province.

	L (*n* = 10)	W (*n* = 10)	H (*n* = 10)	AL (*n* = 10)	AW (*n* = 10)
*Salto Encantado*	2.78 ± 0.39 (2.19–3.42)	2.25 ± 0.27 (1.80–2.69)	1.60 ± 0.26 (1.23–2.02)	1.86 ± 0.36 (1.44–2.74)	1.63 ± 0.27 (1.32–2.18)
*Salto Capioví*	3.29 ± 0.62 (2.64–4.59)	2.61 ± 0.38 (2.16–3.32)	1.46 ± 0.21 (1.17–1.79)	1.89 ± 0.14 (1.75–2.12)	1.64 ± 0.15 (1.41–1.89)
*Salto Chávez*	2.35 ± 0.44 (1.85–3.15)	1.88 ± 0.45 (1.41–2.88)	1.18 ± 0.26 (0.86–1.68)	1.52 ± 0.26 (1.16–1.93)	1.36 ± 0.30 (0.99–1.86)
*Salto Teodoro Cuenca*	2.73 ± 0.42 (2.16–3.53)	2.18 ± 0.36 (1.69–2.81)	1.50 ± 0.31 (1.18–2.16)	1.90 ± 0.41 (1.34–2.74)	1.66 ± 0.35 (1.22–2.37)
*Salto Krysiuk*	2.72 ± 0.41 (2.31–3.58)	2.20 ± 0.37 (1.88–2.95)	1.27 ± 0.21 (1.02–1.73)	1.86 ± 0.26 (1.60–2.30)	1.57 ± 0.20 (1.36–2.01)
*Salto Paca*	3.19 ± 0.40 (2.65–3.82)	2.58 ± 0.30 (2.13–2.90)	1.71 ± 0.29 (1.36–2.19)	2.09 ± 0.31 (1.66–2.72)	1.78 ± 0.23 (1.50–2.24)

Measurements in mm.

AL, aperture length; AW, aperture width; H, height; L, length; W, width.

**Table 3:** Conchological variability of *Acrorbis petricola* from six localities in the Misiones Province and the type locality in Brazil.

	Salto Encantado	Salto Capioví	Salto Chávez	Salto Teodoro Cuenca	Salto Krysiuk	Salto Paca	Nova Teutônia (type locality)*
*Number of whorls*	up to 2 ⅞	up to 2 ¾	up to 2 ½	up to 2 ⅞	up to 2 ¾	up to 2 ⅞	up to 3 ½
*Spiral sculpture*	shell sculptured by spiral lines from faint to coarse ones; some specimens without spiral sculpture	shell sculptured by spiral lines from faint to coarse ones	shell sculptured by spiral lines from faint to coarse ones	shell sculptured by spiral lines from faint to coarse ones	shell sculptured by spiral lines from faint to coarse ones	shell sculptured by spiral lines from faint to coarse ones; some specimens without spiral sculpture	shell sculptured by very weak spiral lines; some specimens without spiral sculpture
*Spire*	elevated to very elevated	elevated	elevated	elevated to very elevated	from low and depressed to elevated	elevated	elevated
*Aperture*	oblique	oblique	oblique	very oblique	from flat to oblique	oblique	very oblique
*Umbilicus*	widely umbilicated; umbilicus from partially to fully covered by a basal reflection of the lip in some specimens	widely umbilicated; umbilicus only partially covered by a basal reflection of the lip in some specimens	widely umbilicated; umbilicus only partially covered by a basal reflection of the lip in some specimens	widely umbilicated; umbilicus only partially covered by a basal reflection of the lip in some specimens	widely umbilicated; umbilicus from partially to fully covered by a basal reflection of the lip in some specimens	widely umbilicated; umbilicus only partially covered by a basal reflection of the lip in some specimens	widely umbilicated; umbilicus covered to a variable extent by a basal reflection of the lip in some specimens

*Conchological characteristics based on Odhner [15], Baker [17], Paraense & Deslandes [19] and Paraense [20,22].

**Fig 6:**
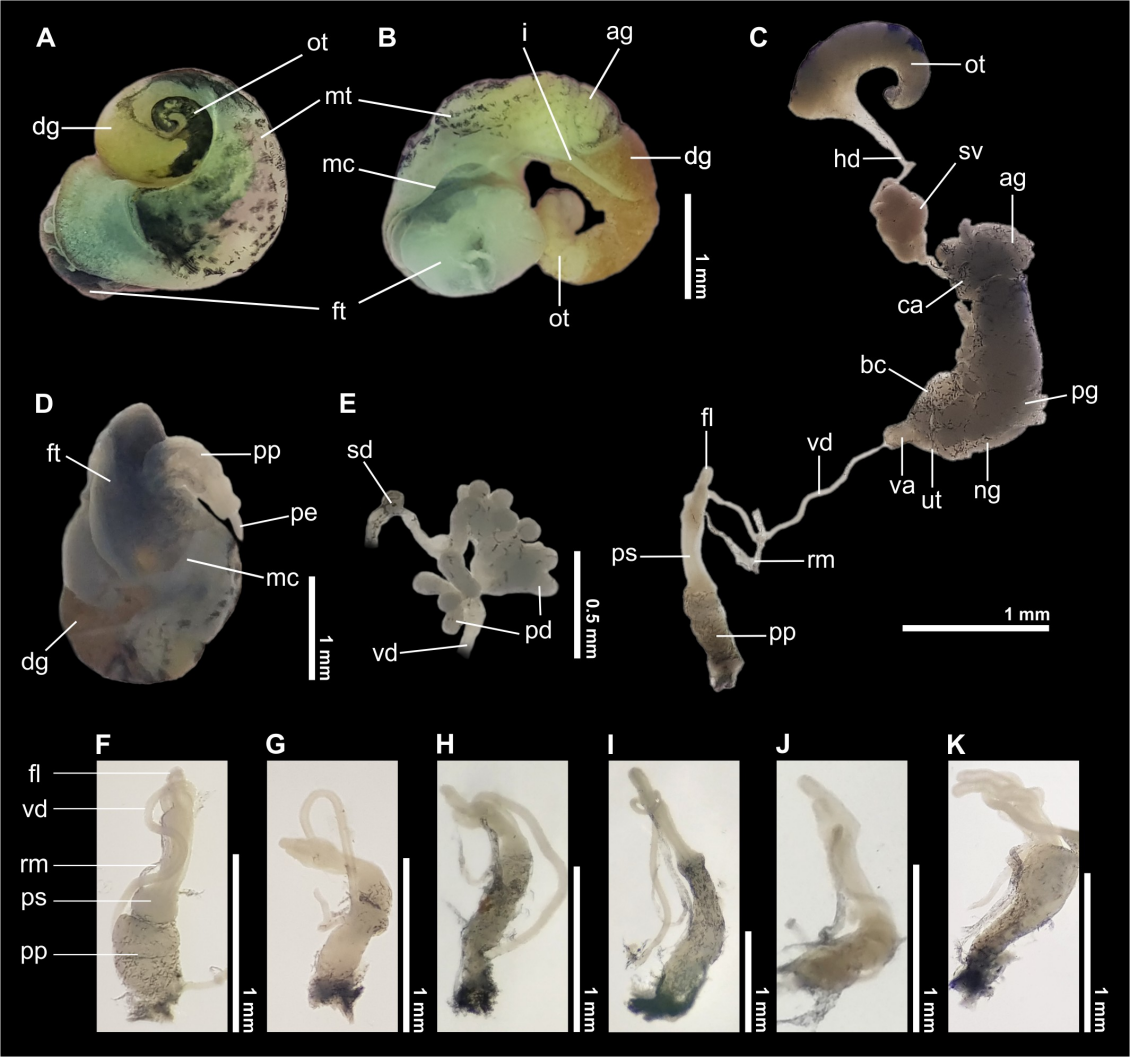
Anatomy of *Acrorbis petricola* from Misiones Province, Argentina. A, B, specimen extracted from the shell in dorsal (A) and ventral (B) views. C, general view of the reproductive system. D, detail of a specimen with penis evaginated in anteroventral view. E, detail of prostatic diverticula. F–K, views of the penial complex from one specimen of each locality examined: F, Salto Encantado; G, Salto Capioví; H, Salto Chávez; I, Salto Teodoro Cuenca; J, Salto Krysiuk; K, Salto Paca. Abbreviatures: ag, albumen gland; bc, bursa copulatrix; ca, carrefour; dg, digestive gland; fl, flagella; ft, foot; hd, hermaphroditic duct; i, intestine; mc, mantle collar; mt, mantle; ng, nidamental gland; ot, ovotestis; pd, prostatic diverticula; pe, penis; pg, prostatic gland; pp, praeputium; ps, penis sheath; rm, retractor muscles of the penial complex; sd, spermiduct; sv, seminal vesicle; ut, uterus; va, vagina; vd, vas deferens.

Reproductive system: the complete genital system is shown in Fig 6. The following distinctive characters are emphasized: ovotestis multilobed with a single row of up to 10 simple unbranched diverticula. Seminal vesicle well-developed, convoluted, at the middle portion of hermaphroditic duct (Fig 6C). Prostate composed of long, slender, unbranched, finger-shaped diverticula; 6–12 diverticula in the main series converging into spermiduct in a fan-like arrangement, 3–4 diverticula protruding into distal spermiduct from opposite direction (Fig 6E). Spermiduct short, highly sinuous. Vas deferens long, inserting at proximal portion of penis sheath. Penis sheath distinctly narrower than praeputium with two flagella at proximal end. Flagella slender, finger-shaped, from ⅓ to about same length as penis sheath; several specimens with flagella unequal in length, asymmetrical and/or widened towards insertion into penis sheath (Fig 6F–6K). Penis unarmed, slender, acicular, longer than the penis sheath (Fig 6D). Praeputium with some tendency to intussusception, separated from penis sheath by a diaphragm. Oviduct short, highly sinuous. Nidamental gland proximally expanded, with numerous small digitiform protuberances at the proximal end. Vagina short, tubular. Bursa copulatrix rounded to pear-shaped. Morphological variability observed between the locations examined and that described for the type locality is presented in Table 4.

**Table 4:** Morpho-anatomical variability in the genital system of *Acrorbis petricola* from six localities in the Misiones Province and the type locality in Brazil.

	Salto Encantado	Salto Capioví	Salto Chávez	Salto Teodoro Cuenca	Salto Krysiuk	Salto Paca	Nova Teutônia (type locality)*
*Prostatic diverticula*	finger-shaped, unbranched, 8–10 in the main series, 3–4 from the opposite direction	finger-shaped, unbranched, 6 in the main series, 4 from the opposite direction	finger-shaped, unbranched, 8 in the main series, 4 from the opposite direction	finger-shaped, unbranched, 7 in the main series, 4 from the opposite direction	finger-shaped, unbranched, 6–8 in the main series, 3–4 from the opposite direction	finger-shaped, unbranched, 12 in the main series, 3–4 from the opposite direction	finger-shaped, unbranched, 5–16 in the main series, 1–3 from the opposite direction
*Penis sheath*	distinctively narrower than praeputium, about ½ to ¾ the length of praeputium	distinctively narrower than praeputium, about ½ to ⅔ the length of praeputium	distinctively narrower than praeputium, about ⅖ to ½ the length of praeputium	distinctively narrower than praeputium, about ⅓ to ⅔ the length of praeputium	distinctively narrower than praeputium, about ½ the length of praeputium	distinctively narrower than praeputium, about ⅓ to ½ the length of praeputium	distinctively narrower than praeputium, about ⅕ to ½ the length of praeputium
*Flagella*	finger-shaped, slender, ⅓ to about same length as penis sheath; some specimens with flagella unequal in length, asymmetrical and widened towards insertion into penis sheath	finger-shaped, slender, ⅓ to about same length as penis sheath; some specimens with flagella unequal in length, asymmetrical	finger-shaped, slender, ½ to about the same length as penis sheath; some specimens with flagella unequal in length, asymmetrical	finger-shaped, slender, ⅓ to ½ the length of penis sheath	finger-shaped, slender, ⅓ to ⅔ the length of penis sheath; some specimens with flagella unequal in length, asymmetrical and widened towards insertion into penis sheath	finger-shaped, slender, ⅘ to about same length as penis sheath; some specimens with flagella widened towards insertion into penis sheath	finger-shaped, ⅓ to 2 times the length of penis sheath
*Bursa copulatrix*	pear-shaped, medium size in relation to nidamental gland	rounded, small size in relation to nidamental gland	rounded to pear-shaped, small size in relation to nidamental gland	pear-shaped, medium size in relation to nidamental gland	pear-shaped, medium size in relation to nidamental gland	rounded, medium to large size in relation to nidamental gland	rounded to pear-shaped, small to large size in relation to nidamental gland

*Morphological features based on Odhner [15], Baker [17], Paraense & Deslandes [19] and Paraense [20].

### Sequence data and phylogenetic analyses

**Fig 7:**
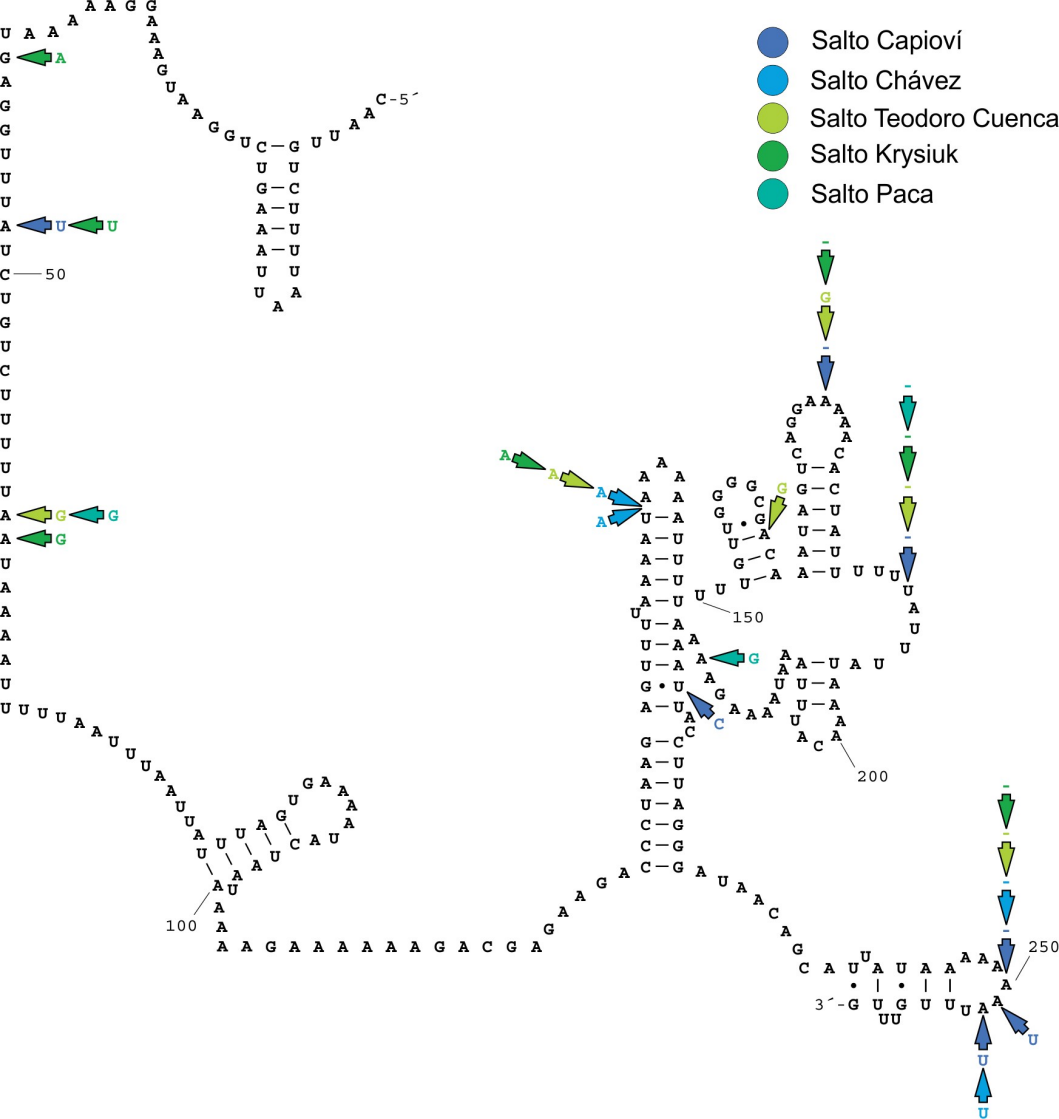
Secondary structure model of domains IV and V of the *16S* gene for *Acrorbis petricola*. **Salto Encantado is shown as reference structure.** Mutational changes for haplotypes from other locations are indicated by colour arrows.

Both mitochondrial genes were successfully amplified in *Acrorbis* specimens from most of the localities, except for the museum material from Puerto Iguazú and Nova Teutônia where amplification of the *16S* and *COI* markers was not possible. Partial *COI* sequences were of 646 bp in length for all individuals, and partial *16S* sequences ranged between 257 and 261 bp. No genetic variation was detected within each waterfall environment. A single unique haplotype per locality per mitochondrial marker was recognised, resulting in a total of six haplotypes per locus. Mean genetic diversity recorded for both markers was high (*COI*: *H*_d_ = 0.862 ± 0.018, π = 0.01050 ± 0.00080; *16S*: *H*_d_ = 0.862 ± 0.018, π = 0.01208 ± 0.00103). Sequence alignments showed a total of 21 and 14 variable positions for the *COI* and *16S* genes, respectively (S3 and S4 Tables). Nucleotide composition of both genes is given in S5 Table. For *COI* haplotypes stop codons were absent, and an ORF = +2 was identified. The secondary structure of the *16S* gene was conserved among the different localities, with polymorphic sites being recorded mostly in unpaired regions or involving alternative basepairing in stems without affecting structural motifs (Fig 7). Genetic distances among *COI* and *16S* haplotypes are summarised in Table 5.

**Table 5:** p-distances between *Acrorbis petricola* haplotypes based on the *COI* (below the diagonal) and *16S* (above the diagonal) genes.

		1	2	3	4	5	6
*Salto Encantado*	**1**	–	0.015564	0.003861	0.011628	0.011673	0.007722
*Salto Capioví*	**2**	0.006192	–	0.011673	0.023346	0.019455	0.023346
*Salto Chávez*	**3**	0.013932	0.013932	–	0.015444	0.015504	0.011628
*Salto Teodoro Cuenca*	**4**	0.007740	0.007740	0.018576	–	0.019380	0.011628
*Salto Krysiuk*	**5**	0.012384	0.009288	0.017028	0.013932	–	0.019455
*Salto Paca*	**6**	0.009288	0.009288	0.020124	0.007740	0.015480	–

**Fig 8:**
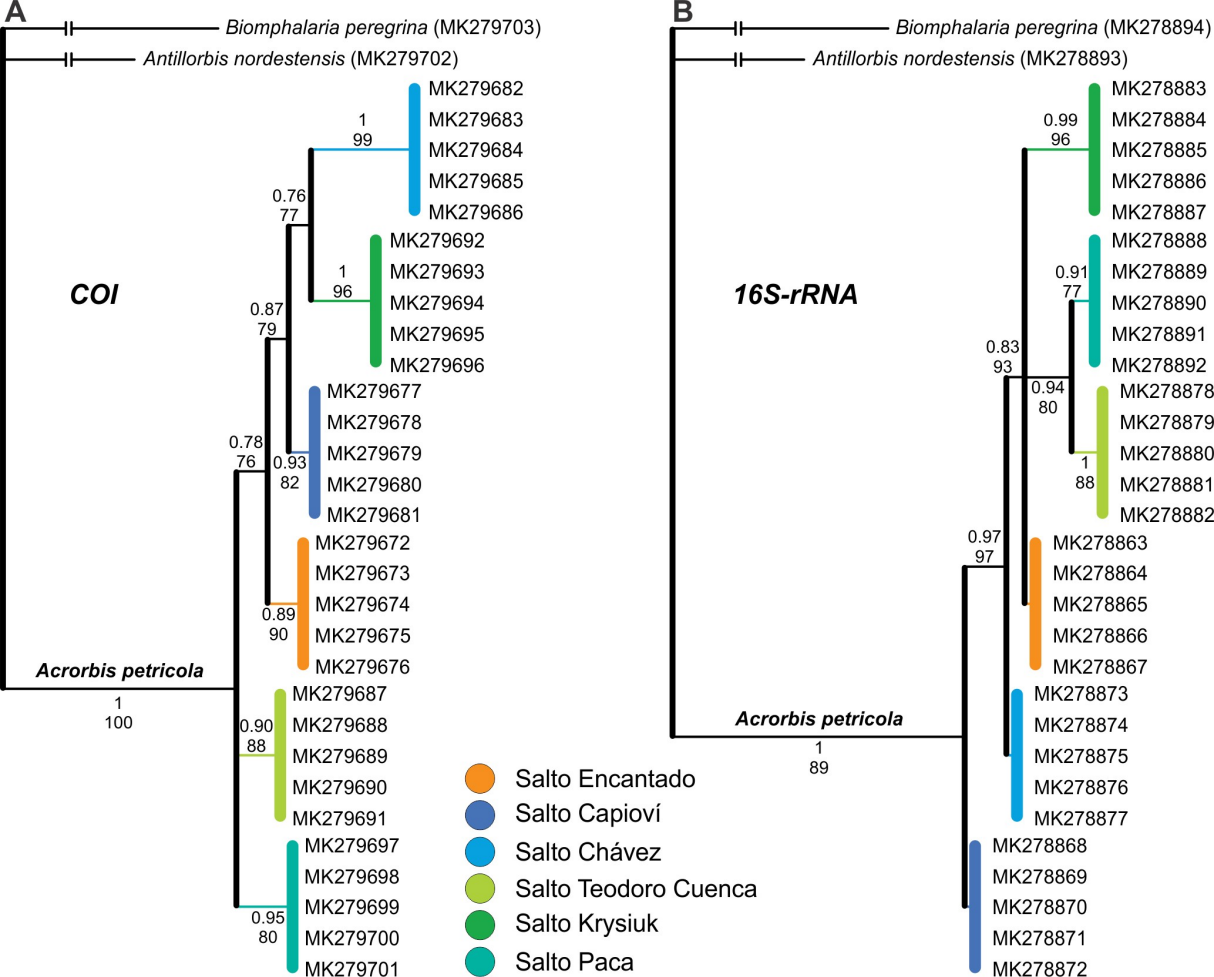
**Bayesian consensus trees of *Acrorbis* specimens from the Misiones Province based on the partial *COI* (A) and *16S* genes (B).** Numbers associated with nodes represent posterior probabilities (BI) and bootstrap values (ML). Numbers within groups are GenBank accession numbers. References to localities are given in colours.

**Fig 9:**
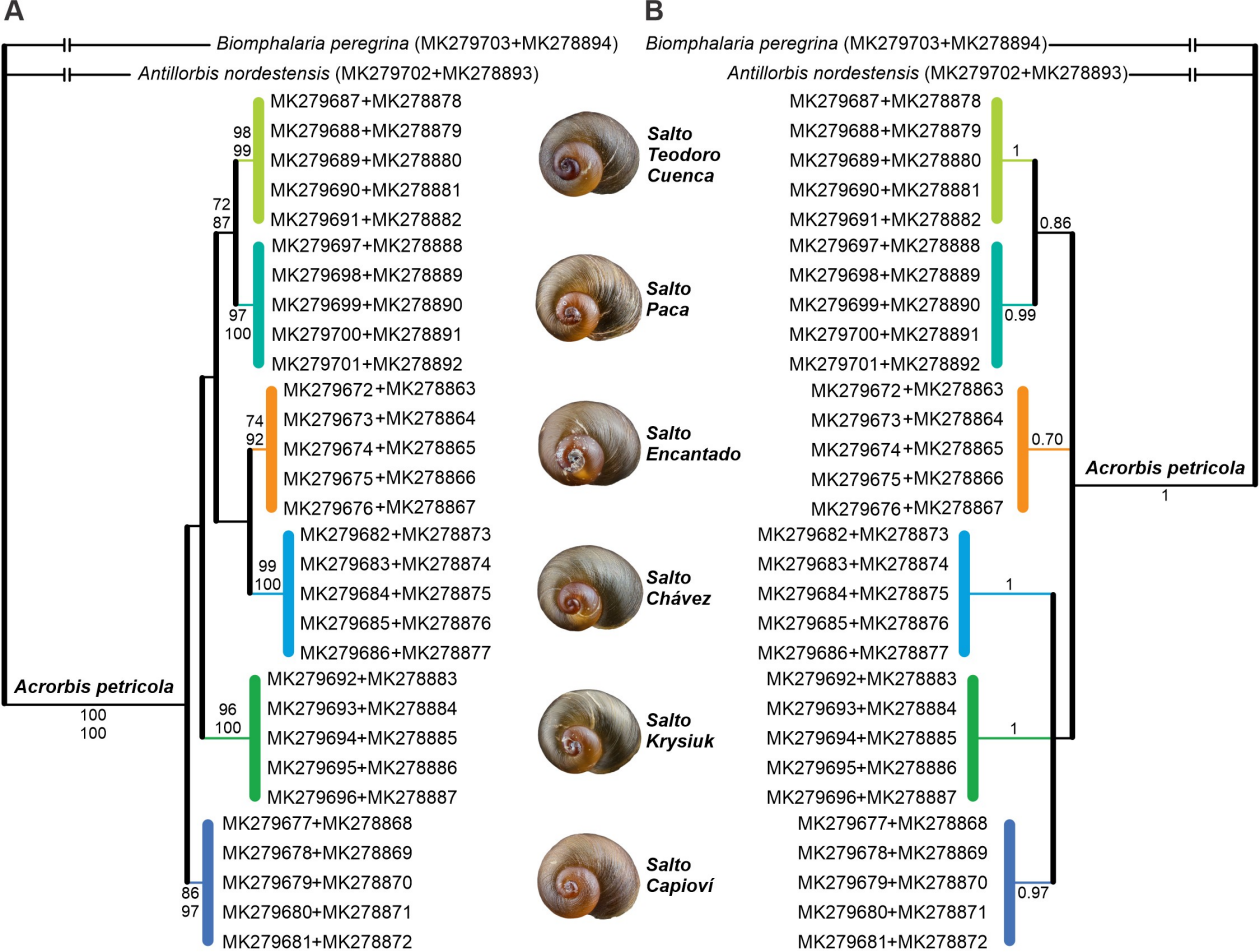
Phylogenetic trees of *Acrorbis* specimens from the Misiones Province based on the concatenated dataset (*COI* + *16S*). A, Maximum-likelihood (ML) tree. B, Bayesian consensus tree. Numbers associated with nodes represent bootstrap values (ML) and posterior probabilities (BI). Support values from PhyML (top) and RAxML (bottom) analyses are shown in ML tree. Numbers within groups are GenBank accession numbers.

**Fig 10:**
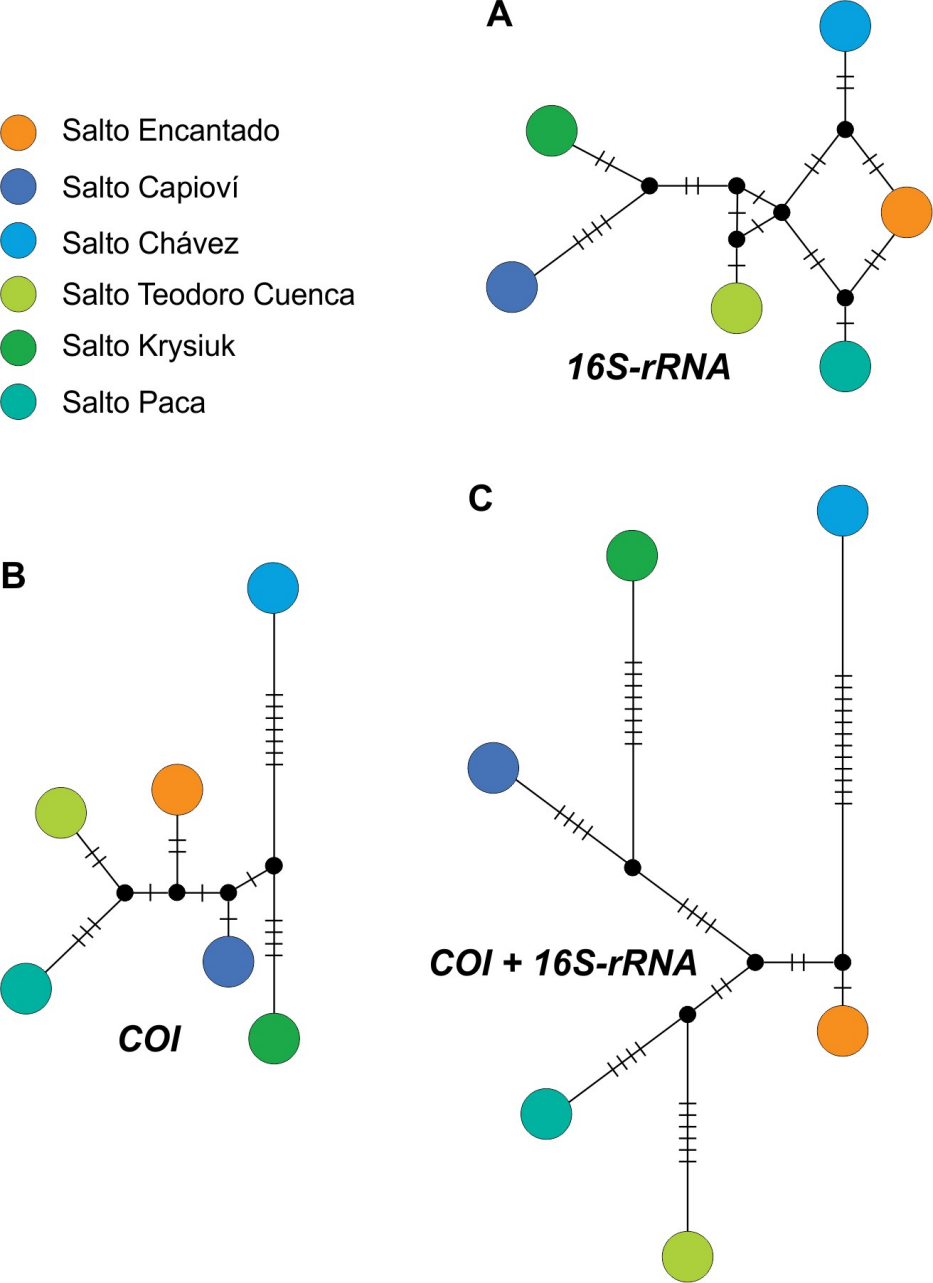
**Median-joining networks of *Acrorbis* haplotypes based on *16S* (A), *COI* (B) and the concatenated dataset (C).** The coloured circles represent individual haplotypes with their size proportional to the number of individuals. Small lines indicate the number of mutations separating haplotypes. Small black circles represent undetected/hypothetical intermediate haplotypes.

Phylogenetic analyses recovered all *Acrorbis petricola* sequences as a well-supported monophyletic group (Figs 8 and 9). In all tree topologies, six phylogenetic lineages for the species were recognized, each one corresponding to a waterfall environment. However, the relationships among these lineages differed between the markers, and could not be resolved through the analysis of the individual regions nor from the concatenated dataset (Figs 8 and 9). Similarly, the organization of haplotype networks differed depending on the marker examined (Fig 10).

## Discussion

In this study, we explored the morphology and genetic background of one the two historical populations of *A*. *petricola* in the Misiones Province, Argentina. In addition, we report for the first time five new populations of the species in southern Upper-Paraná Atlantic Forest, exclusively found on waterfalls from the Misiones Province, which were also morphologically and molecularly characterized. The new records increase the known distribution of *Acrorbis* to both the west and south of the Upper-Paraná Atlantic Forest, with the westernmost record located in Salto Krysiuk and the southernmost record located in Salto Paca. It is worth to mention that the occurrence of the genus *Acrorbis* was reported in highly oxygenated freshwater habitats near the Yacyretá–Apipé rapids in the High Paraná River at the border area between Argentina and Paraguay in the 1980s [10,51]. Such an occurrence, at about 150 km to the west from Salto Krysiuk, would imply a wider distribution of the genus westward. Nonetheless, the shells collected by Rumi [51] supporting that record are unavailable and probably lost. In addition, the Yacyretá–Apipé rapids have disappeared because of damming and the filling up of the Yacyretá Reservoir in the early 1990s [10,12], thus hampering a reliable characterization of that record that remains enigmatic to us.

Morphologically, the specimens of the six populations from the Misiones Province showed intra- and interpopulation conchological variability, mostly in relation to spire and spiral sculpture, which falls within the variation documented for *A*. *petricola* in its type locality [19,20]. For the historical population from Salto Encantado, we found specimens that fit the conchological description of Hylton Scott [21], who refers specimens without spiral sculpture and with an umbilicus partially covered by a brief reflection of the inner lip. However, for that location we also observed specimens with a marked spiral sculpture and a fully covered umbilicus, indicating a greater conchological variability than previously reported. The shells of specimens from Salto Capioví, Salto Chávez, Salto Teodoro Cuenca and Salto Paca were similar to those recorded in Salto Encantado; in contrast, Salto Krysiuk exhibited the most extreme shell configurations in which depressed shells with low spires were observed, resembling those described for the Iguazú National Park [24]. Nonetheless, PCA exhibited no clear geographic separation among the populations in the morphometric space.

The general morphology of the reproductive system of the specimens from the six records examined here is in good agreement with previous studies of Paraense & Deslandes [19] and Paraense [20] for *A*. *petricola*. The penis sheath, flagella, prostatic diverticula and bursa copulatrix were identified to be the structures that exhibited the greatest intra- and interpopulation variability. Paraense [20] has shown that the length proportions of organs with muscular tissue such as the penis sheath and praeputium are highly variable in Planorbidae, since beyond phenotypic variation some variability exists as a result of distension of each organ at the time of fixation. Consequently, morphological differentiation based on these structures should be made with caution. In all the dissected material we have found a pair of slender and finger-like flagella showing a great variability in length within and among localities. Although a single flagellum was referred for *A*. *petricola* in the original description [15], based on material from the same locality Hubendick [18] and Paraense & Deslandes [19] confirmed the existence of two flagella in the male genitalia of the individuals from Nova Teutônia, as recorded here for the Argentinian populations. In other Planorbidae species, the length of flagella is generally useful to discriminate between species (e.g. *Drepanotrema* Fischer & Crosse, 1880), although differences found here can be ascribed to intraspecific variation, as the morphology and proportion of flagella are within the intrapopulation variability previously described for specimens from Nova Teutônia [18–20]. In relation to prostatic diverticula, although it was possible to determine the number of diverticula for the six localities examined, it is likely that such a number will vary as more specimens from each locality be examined; thus, the count presented in this study for each waterfall should be regarded as purely indicative and not definitive. In addition, we documented differences in the shape (from spherical to pyriform) and size (from small to large) of the bursa copulatrix within and among localities. This requires further study to evaluate whether there is a relationship between the shape and size of this organ with the reproductive status of the individuals. Despite this, it is worth noting here that the largest bursa copulatrices were observed in specimens from Salto Paca, which presented the largest shells and also exhibited the longest flagella among the material examined.

Although most of the *A*. *petricola* populations may be indistinguishable based on morphological features alone, our study revealed the existence of interpopulation genetic diversity within the species’ limited distribution range, with absence of intrapopulation genetic variation. The six examined locations were genetically distinct with the presence of a single and unique haplotype per locality, which allows each of them to be recognized as a geographic group. Pairwise genetic distances among the six locations showed similar divergence levels for both markers, ranging from 0.6% to 2.0% for *COI*, and from 0.4% to 2.3% for *16S*. Upon considering the historical population of Salto Encantado as a reference for comparison, Salto Capioví was shown to be the location with the smallest genetic distance for the *COI* gene, but was the one with the greatest divergence for the *16S* gene. Conversely, Salto Chávez exhibited the greatest distance for *COI* and the smallest divergence for *16S*, thus suggesting that both markers are probably evolving at different rates among some of the populations, a finding in need of further investigation. Beyond this, the highest divergences found are within the range of intraspecific variability usually reported in Planorbidae (e.g. [34,52,53]). Based on these values, and supported by morphological evidence, all examined specimens are considered here as belonging to *A*. *petricola*. Nevertheless, it is important to note that DNA sequences are not yet available for the species from its typical locality in Nova Teutônia. Although we attempted to examine the genetic background of that location based on historical material, we had no success in recover DNA probably as a consequence of tissue samples age [54]. Thus, further studies focusing on the genetic divergences and evolutionary affinities of Brazilian specimens are required in order to confirm that all populations belong to the single species *A*. *petricola*. On the contrary, the junior synonym *A*. *odhneri* would need to be revalidated for the Argentinian populations.

For both mitochondrial markers, genetic diversity indices showed high values of mean haplotype and nucleotide diversities. When interpreted in light of the spatial distribution of genetic variation, these findings suggest that despite the short geographical distance between some localities, populations have been genetically isolated for a large amount of time with limited gene flow, a condition attributable to stable, geographically subdivided populations having a long evolutionary history [55,56]. By considering that all *A*. *petricola* populations in the Misiones Province were found in waterfall environments, such a pattern seems fully consistent with vicariance events. These could be related to climatic and tectonic factors associated with the geological history and geomorphological evolution of the fluvial systems of Misiones during the Cenozoic, where the hydrographic network was completely different from the current one [57,58]. Similar evidence of microevolutionary differentiation at small spatial scale has already been documented for other South American endemic freshwater snails (e.g. [59,60]). Further research based on a greater number of individuals and loci per locality is required to gain insights into the evolutionary history, and the spatio-temporal framework of the diversification of *Acrorbis* in the Upper-Paraná Atlantic Forest.

In summary, this study not only broadens the known distribution of the species, but also illustrates a hidden diversity of *Acrorbis* that still remains to be discovered in waterfall environments of the Atlantic Forest, which include genetically differentiated populations. The genealogical relationships among haplotypes depict a complex evolutionary history that needs to be unveiled and taken into account for future development of conservation strategies in this endemic genus. Nonetheless, the identification of six geographically isolated evolutionary lineages in the Misiones Province appear to warrant their future recognition as evolutionarily significant units (ESUs) for conservation prioritisation [61–63]. The Misiones Province has one of the largest systems of protected natural areas in Argentina, comprising different categories ranging from the strictest protected areas to the sustainable-use ones [64]. The information provided here is expected to contribute to the development of future conservation strategies aimed at preserving the gene pool of *Acrorbis* based on the territorial network of protected areas of Misiones. To date, only Salto Encantado and Salto Capioví populations are included within protected areas, in a provincial and municipal park, respectively. In this context, the finding of *Acrorbis* populations in the waterfalls known as Salto Chávez, Salto Teodoro Cuenca, Salto Krysiuk and Salto Paca, widely used as ecotourism resources, could serve as the basis for the environmental protection of such areas, as they harbour a unique genetic diversity of *A*. *petricola*, which is documented here for the first time.

## Supporting information

S1 Table Shell measurements of *Acrorbis petricola* from six localities in the Misiones Province. (DOCX)

S2 Table Component loadings and relative contribution for the five principal components (PCs) obtained by the principal component analysis (PCA). (DOCX)

S3 Table Polymorphic positions of the *COI* gene for *Acrorbis petricola* haplotypes from the Misiones Province. (DOCX)

S4 Table Polymorphic positions of the *16S* gene for *Acrorbis petricola* haplotypes from the Misiones Province. (DOCX)

S5 Table Nucleotide composition of the *COI* and *16S* haplotypes found in *Acrorbis* specimens. (DOCX)
