# Supplementary material for: Hidden diversity in waterfall environments: The genus Acrorbis (Gastropoda: Planorbidae) from the Upper-Paraná Atlantic Forest
Source: PLoS One. 2019 Jul 19;14(7):e0220027. doi: 10.1371/journal.pone.0220027 (PMC6641205; doi:10.1371/journal.pone.0220027)
Supplement: S1 Table — (DOCX) [file pone.0220027.s001.docx]

**S1 Table. Shell measurements of *Acrorbis petricola* from six localities in the Misiones Province.**

| **Locality** | **Voucher** | **L** | **W** | **H** | **AL** | **AW** | **NW** |
| --- | --- | --- | --- | --- | --- | --- | --- |
| *Salto Encantado* | IBS-Ma 376-1 | 3.42 | 2.69 | 2.02 | 1.82 | 1.84 | 2 ⅞ |
|  | IBS-Ma 376-2 | 2.58 | 2.20 | 1.34 | 1.67 | 1.45 | 2 ½ |
|  | IBS-Ma 376-3 | 2.42 | 1.97 | 1.60 | 1.72 | 1.45 | 2 ⅞ |
|  | IBS-Ma 376-5 | 3.01 | 2.53 | 1.88 | 2.07 | 1.83 | 2 ¾ |
|  | IBS-Ma 376-6 | 2.52 | 2.09 | 1.32 | 1.60 | 1.35 | 2 ¾ |
|  | IBS-Ma 376-7 | 3.14 | 2.33 | 1.63 | 1.91 | 1.74 | 2 ¾ |
|  | IBS-Ma 376-8 | 2.52 | 2.11 | 1.54 | 1.69 | 1.45 | 2 ¼ |
|  | IBS-Ma 376-9 | 3.13 | 2.49 | 1.89 | 2.74 | 2.18 | 2 ¾ |
|  | IBS-Ma 376-10 | 2.19 | 1.80 | 1.23 | 1.44 | 1.32 | 2 ⅜ |
|  | IBS-Ma 376-15 | 2.87 | 2.29 | 1.54 | 1.93 | 1.65 | 2 ¾ |
| *Salto Capioví* | IBS-Ma 377-1 | 2.81 | 2.35 | 1.46 | 1.79 | 1.56 | 2 ¾ |
|  | IBS-Ma 377-3 | 2.90 | 2.48 | 1.22 | 1.75 | 1.64 | 2 ½ |
|  | IBS-Ma 377-5 | 3.06 | 2.62 | 1.40 | 1.92 | 1.76 | 2 ¾ |
|  | IBS-Ma 377-6 | 2.68 | 2.16 | 1.31 | 1.77 | 1.41 | 2 ⅜ |
|  | IBS-Ma 377-7 | 3.22 | 2.56 | 1.69 | 2.12 | 1.65 | 2 ⅜ |
|  | IBS-Ma 377-8 | 2.64 | 2.16 | 1.17 | 1.76 | 1.45 | 2 ⅜ |
|  | IBS-Ma 377-9 | 3.35 | 2.63 | 1.65 | 2.10 | 1.89 | 2 ⅜ |
|  | IBS-Ma 377-10 | 3.93 | 3.14 | 1.30 | 1.91 | 1.62 | 2 ⅜ |
|  | IBS-Ma 377-11 | 4.59 | 3.32 | 1.58 | 1.78 | 1.62 | 2 ¼ |
|  | IBS-Ma 377-12 | 3.69 | 2.69 | 1.79 | 2.01 | 1.83 | 2 ¼ |
| *Salto Chávez* | IBS-Ma 378-1 | 3.15 | 2.88 | 1.68 | 1.93 | 1.86 | 2 ½ |
|  | IBS-Ma 378-2 | 2.09 | 1.52 | 0.88 | 1.63 | 1.35 | 2 ¼ |
|  | IBS-Ma 378-4 | 2.04 | 1.63 | 1.13 | 1.33 | 1.18 | 2 ½ |
|  | IBS-Ma 378-5 | 1.98 | 1.66 | 1.09 | 1.29 | 1.13 | 2 ½ |
|  | IBS-Ma 378-6 | 2.20 | 1.67 | 1.11 | 1.35 | 1.17 | 2 ⅜ |
|  | IBS-Ma 378-7 | 1.85 | 1.41 | 0.96 | 1.16 | 0.99 | 2 ⅜ |
|  | IBS-Ma 378-8 | 2.06 | 1.60 | 0.86 | 1.42 | 1.15 | 2 ⅜ |
|  | IBS-Ma 378-11 | 2.56 | 2.00 | 1.26 | 1.52 | 1.83 | 2 ⅜ |
|  | IBS-Ma 378-12 | 2.96 | 2.35 | 1.40 | 1.90 | 1.55 | 2 ⅜ |
|  | IBS-Ma 378-13 | 2.57 | 2.04 | 1.42 | 1.68 | 1.41 | 2 ¼ |
| *Salto Teodoro Cuenca* | IBS-Ma 379-1 | 2.22 | 1.92 | 1.27 | 1.61 | 1.35 | 2 ⅜ |
|  | IBS-Ma 379-3 | 3.26 | 2.76 | 2.16 | 2.11 | 1.92 | 2 |
|  | IBS-Ma 379-4 | 2.16 | 1.69 | 1.18 | 1.33 | 1.22 | 2 ⅜ |
|  | IBS-Ma 379-8 | 2.70 | 2.16 | 1.31 | 1.73 | 1.44 | 2 ⅜ |
|  | IBS-Ma 379-10 | 2.58 | 1.97 | 1.28 | 1.65 | 1.39 | 2 ¼ |
|  | IBS-Ma 379-22 | 2.60 | 2.03 | 1.33 | 1.75 | 1.61 | 1 ⅞ |
|  | IBS-Ma 379-27 | 2.84 | 2.16 | 1.49 | 2.34 | 1.92 | 2 ⅞ |
|  | IBS-Ma 379-29 | 2.79 | 2.25 | 1.62 | 2.05 | 1.84 | 2 ¾ |
|  | IBS-Ma 379-31 | 3.53 | 2.81 | 1.86 | 2.74 | 2.37 | 2 ¾ |
|  | IBS-Ma 379-35 | 2.61 | 2.01 | 1.56 | 1.66 | 1.50 | 2 ¾ |
| *Salto Krysiuk* | IBS-Ma 380-3 | 2.48 | 2.06 | 1.28 | 1.60 | 1.40 | 2 ¾ |
|  | IBS-Ma 380-4 | 2.56 | 1.98 | 1.09 | 1.66 | 1.56 | 2 ¾ |
|  | IBS-Ma 380-6 | 2.44 | 1.89 | 1.24 | 1.68 | 1.50 | 2 ¼ |
|  | IBS-Ma 380-7 | 3.21 | 2.73 | 1.41 | 2.22 | 1.75 | 2 ½ |
|  | IBS-Ma 380-8 | 2.54 | 2.01 | 1.16 | 2.08 | 1.68 | 2 ¼ |
|  | IBS-Ma 380-12 | 2.31 | 1.88 | 1.09 | 1.80 | 1.42 | 2 ¼ |
|  | IBS-Ma 380-14 | 2.38 | 1.93 | 1.02 | 1.67 | 1.36 | 2 ¼ |
|  | IBS-Ma 380-20 | 2.72 | 2.22 | 1.25 | 1.67 | 1.46 | 2 ⅜ |
|  | IBS-Ma 380-33 | 3.58 | 2.95 | 1.73 | 2.30 | 2.01 | 2 ¾ |
|  | IBS-Ma 380-35 | 2.98 | 2.34 | 1.41 | 1.94 | 1.58 | 2 ½ |
| *Salto Paca* | IBS-Ma 381-1 | 3.10 | 2.44 | 1.75 | 2.36 | 1.96 | 2 ¾ |
|  | IBS-Ma 381-2 | 3.32 | 2.66 | 1.84 | 1.89 | 1.85 | 2 ⅞ |
|  | IBS-Ma 381-3 | 3.47 | 2.82 | 1.72 | 2.01 | 1.92 | 2 ⅞ |
|  | IBS-Ma 381-4 | 2.65 | 2.13 | 1.36 | 1.66 | 1.50 | 2 ½ |
|  | IBS-Ma 381-5 | 3.38 | 2.87 | 1.74 | 2.72 | 2.24 | 2 ¾ |
|  | IBS-Ma 381-6 | 3.57 | 2.90 | 2.14 | 2.32 | 1.88 | 2 ¾ |
|  | IBS-Ma 381-7 | 2.67 | 2.26 | 1.53 | 1.98 | 1.57 | 2 ⅜ |
|  | IBS-Ma 381-8 | 2.72 | 2.19 | 1.38 | 1.81 | 1.56 | 2 ⅞ |
|  | IBS-Ma 381-9 | 3.16 | 2.61 | 1.48 | 1.96 | 1.58 | 2 ⅜ |
|  | IBS-Ma 381-10 | 3.82 | 2.89 | 2.19 | 2.15 | 1.75 | 2 ¼ |

Measurements in mm.

AL, aperture length; AW, aperture width; H, height; L, length; NW, number of whorls; W, width.
