# Supplementary material for: Hidden diversity in waterfall environments: The genus Acrorbis (Gastropoda: Planorbidae) from the Upper-Paraná Atlantic Forest
Source: PLoS One. 2019 Jul 19;14(7):e0220027. doi: 10.1371/journal.pone.0220027 (PMC6641205; doi:10.1371/journal.pone.0220027)
Supplement: S2 Table — (DOCX) [file pone.0220027.s002.docx]

**S2 Table. Component loadings and relative contribution for the five principal components (PCs) obtained by the principal component analysis (PCA).**

|  | **PC1**  **(84.80%)** | **PC2**  **(7.01%)** | **PC3**  **(6.26%)** | **PC4**  **(1.36%)** | **PC5**  **(0.56%)** |
| --- | --- | --- | --- | --- | --- |
| L | 0.44940 | -0.37949 | 0.40885 | -0.00466 | 0.69775 |
| W | 0.46350 | -0.35980 | 0.37624 | -0.05362 | -0.71503 |
| H | 0.49364 | -0.26135 | -0.82543 | -0.07849 | 0.02306 |
| AL | 0.40844 | 0.65600 | 0.09702 | -0.62638 | 0.03268 |
| AW | 0.41558 | 0.47737 | 0.02336 | 0.77368 | -0.01655 |

AL, aperture length; AW, aperture width; H, height; L, length; W, width.
