# Supplementary material for: Hidden diversity in waterfall environments: The genus Acrorbis (Gastropoda: Planorbidae) from the Upper-Paraná Atlantic Forest
Source: PLoS One. 2019 Jul 19;14(7):e0220027. doi: 10.1371/journal.pone.0220027 (PMC6641205; doi:10.1371/journal.pone.0220027)
Supplement: S3 Table — (DOCX) [file pone.0220027.s003.docx]

**S3 Table. Polymorphic positions of the *COI* gene for *Acrorbis petricola* haplotypes from the Misiones Province.**

|  | **34** | **40** | **56** | **58** | **84** | **136** | **172** | **196** | **220** | **268** | **283** | **286** | **313** | **322** | **328** | **337** | **367** | **472** | **496** | **511** | **640** |
| --- | --- | --- | --- | --- | --- | --- | --- | --- | --- | --- | --- | --- | --- | --- | --- | --- | --- | --- | --- | --- | --- |
| *Salto Encantado* | G | T | T | G | C | C | G | A | A | T | A | G | A | A | A | C | T | T | T | A | T |
| *Salto Capioví* | A | · | · | · | · | · | · | · | · | · | · | · | · | · | · | T | C | · | · | G | · |
| *Salto Chávez* | · | · | C | · | · | T | · | · | G | · | · | A | G | G | · | T | · | · | · | G | C |
| *Salto Teodoro Cuenca* | A | · | · | A | · | · | A | · | · | · | · | · | · | · | G | · | · | · | · | G | · |
| *Salto Krysiuk* | A | A | · | · | T | T | · | G | · | · | · | · | · | · | · | T | · | · | C | G | · |
| *Salto Paca* | A | · | · | A | · | · | · | · | · | C | G | · | · | · | · | · | · | C | · | G | · |

Numbers indicate the position of variable sites. Salto Encantado is shown as reference sequence; dot indicates identity with the reference sequence.
