# Supplementary material for: Hidden diversity in waterfall environments: The genus Acrorbis (Gastropoda: Planorbidae) from the Upper-Paraná Atlantic Forest
Source: PLoS One. 2019 Jul 19;14(7):e0220027. doi: 10.1371/journal.pone.0220027 (PMC6641205; doi:10.1371/journal.pone.0220027)
Supplement: S4 Table — (DOCX) [file pone.0220027.s004.docx]

**S4 Table. Polymorphic positions of the *16S* gene for *Acrorbis petricola* haplotypes from the Misiones Province.**

|  | **41** | **48** | **60** | **61** | **140** | **141** | **164** | **178** | **192** | **218** | **223** | **252** | **253** | **254** |
| --- | --- | --- | --- | --- | --- | --- | --- | --- | --- | --- | --- | --- | --- | --- |
| *Salto Encantado* | G | A | A | A | – | – | A | A | T | A | T | A | A | A |
| *Salto Capioví* | · | T | · | · | – | – | · | – | – | · | C | – | T | T |
| *Salto Chávez* | · | · | · | · | A | A | · | · | · | · | · | – | · | T |
| *Salto Teodoro Cuenca* | · | · | G | · | – | A | G | G | – | · | · | – | · | · |
| *Salto Krysiuk* | A | T | · | G | – | A | · | – | – | · | · | – | · | · |
| *Salto Paca* | · | · | G | · | – | – | · | · | – | G | · | · | · | · |

Numbers indicate the position of variable sites. Salto Encantado is shown as reference sequence; dot indicates identity with the reference sequence; dash represent a gap.
