# Supplementary material for: Hidden diversity in waterfall environments: The genus Acrorbis (Gastropoda: Planorbidae) from the Upper-Paraná Atlantic Forest
Source: PLoS One. 2019 Jul 19;14(7):e0220027. doi: 10.1371/journal.pone.0220027 (PMC6641205; doi:10.1371/journal.pone.0220027)
Supplement: S5 Table — (DOCX) [file pone.0220027.s005.docx]

**S5 Table. Nucleotide composition of the *COI* and *16S* haplotypes found in *Acrorbis* specimens.**

|  | **A** | **C** | **G** | **T** | **AT content** | **GC content** | **Size** |
| --- | --- | --- | --- | --- | --- | --- | --- |
| ***COI*** |  |  |  |  |  |  |  |
| *Salto Encantado* | 159  (24.61%) | 84  (13.00%) | 111  (17.18%) | 292  (45.20%) | 69.81% | 30.19% | 646 pb |
| *Salto Capioví* | 159  (24.61%) | 84  (13.00%) | 111  (17.18%) | 292  (45.20%) | 69.81% | 30.19% | 646 pb |
| *Salto Chávez* | 156  (24.15%) | 84  (13.00%) | 114  (17.65%) | 292  (45.20%) | 69.35% | 30.65% | 646 pb |
| *Salto Teodoro Cuenca* | 160  (24.77%) | 84  (13.00%) | 110  (17.03%) | 292  (45.20%) | 69.97% | 30.03% | 646 pb |
| *Salto Krysiuk* | 159  (24.61%) | 82  (12.69%) | 112  (17.34%) | 293  (45.36%) | 69.97% | 30.03% | 646 pb |
| *Salto Paca* | 159  (24.61%) | 86  (13.31%) | 111  (17.18%) | 290  (44.89%) | 69.50% | 30.50% | 646 pb |
| ***16S*** |  |  |  |  |  |  |  |
| *Salto Encantado* | 116  (44.62%) | 20  (7.69%) | 36  (13.85%) | 88  (33.85%) | 78.46% | 21.54% | 260 pb |
| *Salto Capioví* | 111  (43.19%) | 21  (8.17%) | 36  (14.01%) | 89  (34.63%) | 77.82% | 22.18% | 257 pb |
| *Salto Chávez* | 116  (44.44%) | 20  (7.66%) | 36  (13.79%) | 89  (34.10%) | 78.54% | 21.46% | 261 pb |
| *Salto Teodoro Cuenca* | 113  (43.63%) | 20  (7.72%) | 39  (15.06%) | 87  (33.59%) | 77.22% | 22.78% | 259 pb |
| *Salto Krysiuk* | 114  (44.19%) | 20  (7.75%) | 36  (13.95%) | 88  (34.11%) | 78.29% | 21.29% | 258 pb |
| *Salto Paca* | 114  (44.02%) | 20  (7.72%) | 38  (14.67%) | 87  (33.59%) | 77.61% | 22.39% | 259 pb |
